# Rare incidence of a diffuse brain metastatic carcinoma: A case report

**DOI:** 10.3892/ol.2014.2377

**Published:** 2014-07-24

**Authors:** XIANG SUN, ZHIJUAN CHEN, WEIDONG YANG, FUHUA YU, JINGWANG ZHAO, PING HE, ZENGGUANG WANG

**Affiliations:** Department of Neurosurgery, Tianjin Medical University General Hospital, Tianjin 300052, P.R. China

**Keywords:** brain metastases, metastatic adenocarcinoma, surgery, brain tumor

## Abstract

Brain metastases generally present in the parenchyma of the brain. In the current report, a very rare case of brain metastasis, which simultaneously invaded the subgaleal region, the skull, and the dural and cavernous sinuses is presented. The patient, a 54-year-old female, complained of a progressive headache and exhibited the symptoms of intracranial hypertension. Coronal contrast-enhanced T1-weighted magnetic resonance imaging (MRI) showed high intensity signals in the subgaleal tissue of the left frontoparietal area, as well as in the dural and the cavernous sinuses. The patient was initially diagnosed with an intracranial infection, however, the administered treatment was ineffective. The patient subsequently underwent a biopsy and the pathological diagnosis was determined as a metastatic adenocarcinoma; a primary tumor was not identified during the examinations. Surgical removal of certain metastases and a decompressive craniectomy were performed to relieve the intracranial hypertension. However, the prognosis was unsatisfactory. The patient’s neurological condition progressively worsened and an axial computed tomography scan with a bone window demonstrated a bulging growth in the brain tissue. The patient succumbed after one month due to the widespread metastasis. Thus, this case presents the unusual clinical development of this type of metastatic adenocarcinoma. In addition, due to the intracranial hypertension, the unusual sites of the high intensity signals in the MRI and the lack of a primary tumor, the patient was misdiagnosed with an intracranial infection. Furthermore, this case highlights the necessity for conducting a biopsy as soon as possible and demonstrates the poor prognosis associated with this type of patient.

## Introduction

Brain metastases are a common complication in patients with cancer and are an increasingly important cause of morbidity and mortality (1). They develop in 10–30% of adults and in 6–10% of children with cancer (2–8). In adults, the most common types of primary tumor responsible for brain metastases are lung (50%), breast (15–20%), unknown primary (10–15%), melanoma (10%), and colon (5%) (2,3). In children, the most common source of brain metastases are sarcomas, neuroblastoma, and germ cell tumors (2,9). Metastases from breast, colon, and renal cell carcinoma are frequently single, while melanoma and lung cancer have a greater tendency to produce multiple metastases (2,10). They generally present in the cortex of the frontal, parietal or temporal lobe, however, they rarely invade the skull and meninges (6). The current study, to the best of our knowledge, is the first reported case of brain metastasis which simultaneously invaded the subgaleal region, the skull, and the dural and cavernous sinuses. In general, such patients can easily be misdiagnosed, so the results of this case report may improve clinical studies of this type.

## Case report

**Figure 1:**
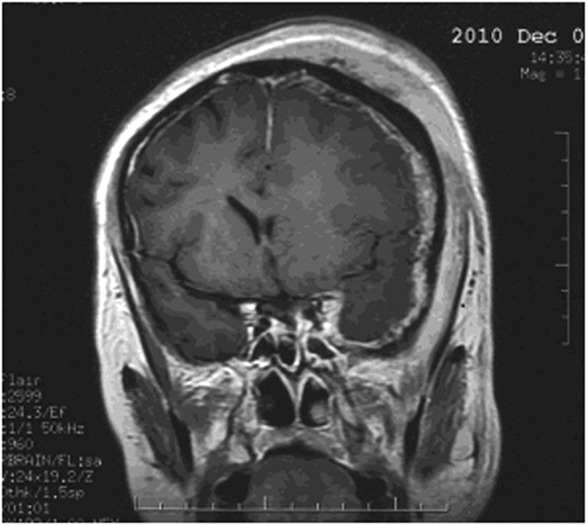
Coronal contrast-enhanced T1-weighted magnetic resonance imaging scan showing a thickening of the soft tissue, which was identified by a high intensity signal in the subgaleal tissue of the left frontoparietal area, and in the dural and cavernous sinuses.

**Figure 2:**
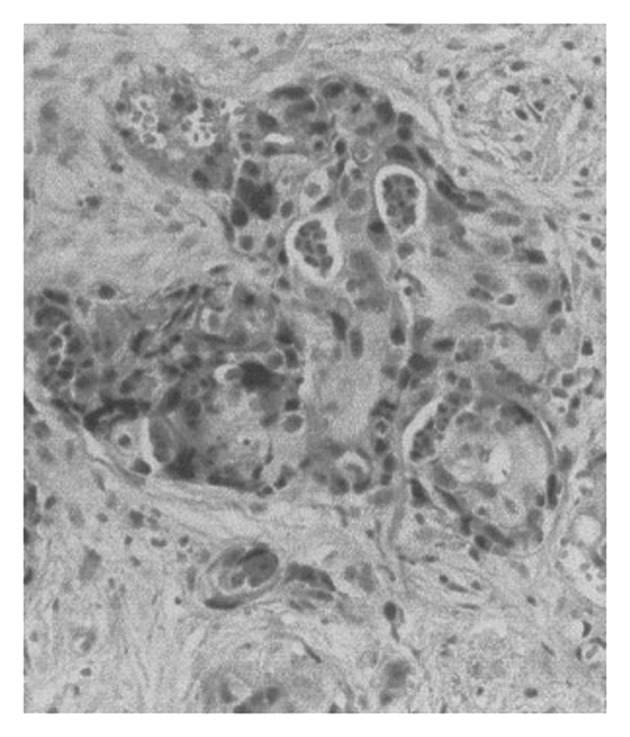
Biopsy revealing the metastatic adenocarcinoma in the left frontal, pariental, and temporal bones and the dural and subgaleal tissue. (Hematoxylin and eosin stain; magnification, ×400).

**Figure 3:**
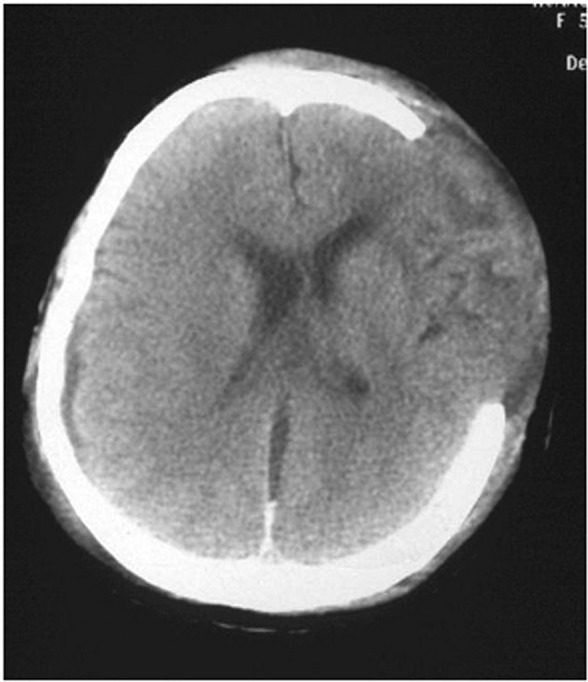
Axial computed tomography scan with a bone window demonstrates a marked bulging of the brain tissue, which caused the mid-line and the ventricular system to shift to the left.

In December 2010, a 54-year-old female presented at the Tianjin Medical University General Hospital (Tianjin, China) with a progressive headache of the left parietal area of the brain that had persisted for one month. The patient provided written informed consent. The results of the patient examination indicated lethargy, double eyelid edema, a 5×7-cm^2^ purple swollen area in the left frontal top scalp with tenderness, hypalgesia of the left frontal scalp, chemosis, eye fixation and a positive Babinski sign on the right side. The cerebrospinal fluid pressure was 250 mmH_2_O, however, no other abnormalities were identified during the physical and laboratory examinations. Coronal contrast-enhanced T1-weighted magnetic resonance imaging (MRI) revealed a soft tissue swelling of the subgaleal tissue of the left frontoparietal area, and a high intensity signal in the dural and cavernous sinuses (Fig. 1). As a result of the observations of the clinical manifestations, the patient was initially diagnosed with an intracranial infection. The patient subsequently underwent a biopsy of the abnormal tissue, which included tissue samples from the skull, dural sinus and subgaleal region. The pathological diagnosis was determined as a metastatic adenocarcinoma (Fig. 2). However, following a series of imaging assessments, the primary tumor was not located. In order to relieve the intracranial hypertension, the patient underwent surgical resection of the majority of the abnormal tissues and received a decompressive craniectomy. The patient’s postoperative course was uneventful and initially the symptoms improved. However, after two weeks, the patient’s neurological condition began to progressively worsen. Axial computed tomography (CT) scan with a bone window demonstrated a bulging growth in the brain tissue, which caused the ventricular system to shift to the left (Fig. 3). Due to the widespread metastasis, the secondary surgical removal had no effect. The patient declined further treatment and succumbed after one month.

## Discussion

According to previous studies, the majority of brain metastases present in the brain parenchyma and rarely invade the skull or the meningeal (11–13). The current case of a patient with a brain metastatic carcinoma invading the subgaleal region, skull, and the dural and cavernous sinuses is considered to be particularly uncommon. In the present case, due to the presence of intracranial hypertension, the sites of high intensity signals in the MRI and the lack of a primary tumor, the patient was misdiagnosed as presenting with an intracranial infection. Therefore, the current case report presents the clinical development of an unusual form of brain metastasis and highlights the necessity for conducting a biopsy as soon as possible in this type of patient.

In conclusion, in the patient described in the present case report, the metastases invaded the subgaleal region, the skull, and the dural and cavernous sinuses simultaneously, which, to the best of our knowledge, has not previously been reported. Although the patient underwent surgery (surgical resection of the majority of the abnormal tissues and a decompressive craniectomy), the patient succumbed after one month, which is consistent with the poor prognosis associated with brain metastases. This patient presented with intracranial hypertension; however, the final diagnosis was determined via pathological examination as a brain metastasis, although the primary tumor was not found during the imaging examinations. Thus, this type of patient may easily be misdiagnosed as exhibiting an intracranial infection, therefore, performance of a biopsy is considered to be necessary in the early stages of the diagnostic procedures. Furthermore, the diagnosis of intracranial malignant metastases must be considered for patients >40-year-old, who present with rapid progression of clinical manifestations, such as a headache and obvious intracranial hypertension, even when no primary tumor is identified. The accurate diagnosis of this type of cancer relies on the results that are obtained via biopsy; however, the prognosis for this type of patient is generally poor.
